# Codon usage bias and molecular evolution of *Dnajc15* across avian lineages

**DOI:** 10.3389/fgene.2026.1841532

**Published:** 2026-06-16

**Authors:** Zeshuo Zhou, Jianke Yang, Duminda S. B. Dissanayake, Lisa Schwanz, Arthur Georges, Lei Xiong

**Affiliations:** 1 School of Basic Medical Sciences, Wannan Medical University, Wuhu, China; 2 Institute for Applied Ecology, University of Canberra, Canberra, ACT, Australia; 3 School of Biological, Earth and Environmental Sciences, Faculty of Science at the University of New South Wales, Sydney, NSW, Australia

**Keywords:** avian, codon usage bias, Dnajc15, HSP40 (Dnajc), molecular evolution

## Abstract

**Background:**

Dnajc15, encoding the methylation-controlled J-protein (MCJ) of the HSP40 family, is an endogenous repressor of the mitochondrial respiratory chain and a key regulator of mitochondrial metabolism. The codon usage patterns and molecular evolution of Dnajc15 in birds remain largely uncharacterized.

**Methods:**

We present the first systematic characterization of Dnajc15 codon usage patterns and adaptive evolution across 27 bird species representative of 11 avian orders, using codon usage bias analysis, PAML-based selection pressure analysis, and gene family analysis.

**Results:**

Codon usage bias analysis revealed a preference for A/U-ending codons and weak overall codon bias (effective codon number: 51.72–61.00). Selection analyses indicated predominant purifying selection (ω = 0.33). The M7 vs. M8 site model identified four candidate positively selected sites (positions 8, 17, 19, and 57), located in the N-terminal region and J-domain of the MCJ protein, though this signal was not corroborated by the M1a vs. M2a comparison, suggesting at most limited or episodic positive selection. Dnajc15 was retained across all examined vertebrate lineages despite widespread contraction of other Dnajc family members.

**Conclusion:**

Dnajc15 is a highly conserved component of the avian mitochondrial regulatory system, with limited evidence for adaptive variation at a small number of candidate sites, consistent with strong long-term functional constraint.

## 1 Introduction

The study of synonymous codon usage extends beyond the primary role of codons in encoding amino acids, and has broad implications for the regulation of gene expression and molecular evolution. Comparative analyses of coding sequences across taxa have revealed distinct patterns of synonymous codon usage among different organisms, reflecting both phylogenetic history and lineage-specific compositional and selective processes (Arella et al., 2021). Birds, with their diverse habitats and life histories, display significant variation in synonymous codon preferences shaped by shared ancestry as well as gene and lineage-specific compositional and selective pressures (Cambray and Mazel, 2008; Cope and Gilchrist, 2022; Hershberg and Petrov, 2008).

Synonymous codon usage is shaped by multiple forces, including mutational pressure, translational selection, and genetic drift. Of the 61 sense codons encoding 20 amino acids, only methionine and tryptophan are specified by single codons; the remaining 18 amino acids are each encoded by two or more synonymous codons, which most commonly differ at the third codon position (Chakraborty et al., 2019; Rao et al., 2011). The observed non-random patterns of codon frequency are influenced by factors including GC content, mutation rate, gene expression level, tRNA abundance, protein structure, and environmental stress (Guan et al., 2018). Among the theoretical frameworks proposed to explain these patterns, the mutation-selection-drift model—which attributes codon usage to the combined action of natural selection, random mutation, and genetic drift—has received increasing empirical support and provides a useful framework for studying codon usage evolution (Bulmer, 1991).

Mitochondrial function and metabolic regulation are critical determinants of cellular homeostasis, and the high aerobic demands of powered flight make birds a particularly compelling system in which to examine the evolutionary constraints acting on mitochondrial regulatory genes. The methylation-controlled J-protein (MCJ), encoded by Dnajc15 of the HSP40 family, is an endogenous repressor of mitochondrial Complex I activity and plays roles in mitochondrial biogenesis, metabolic regulation, and fertilization (Hatle et al., 2013; Jiang et al., 2019). As a functionally critical regulator of the mitochondrial respiratory chain, Dnajc15 is therefore a plausible candidate for strong evolutionary constraint. Birds, with their exceptionally high metabolic demands associated with powered flight, represent a particularly informative system for examining the evolutionary dynamics of mitochondrial regulatory genes. Despite this, the codon usage patterns and molecular evolution of Dnajc15 in birds remain largely uncharacterized.

Previous codon usage bias studies in birds have examined members of the HSP family, including HSP60 (Yang et al., 2021), revealing that selection may contribute substantially to patterns of codon usage in avian heat shock protein genes. However, Dnajc15 differs fundamentally from HSP60 in both protein size (148 vs. ∼573 amino acids), molecular function (respiratory chain inhibitor vs. protein chaperone), and evolutionary origin (Dnajc15 is a vertebrate duplication of Dnajc19 that lacks the N-terminal domain). Whether similar or distinct selective forces operate on Dnajc15 codon usage in birds, and how these forces relate to the long-term functional constraint of the gene across vertebrate lineages, remains an open question.

To address this, we employed a comparative framework combining codon usage bias analysis, selection pressure analysis, and gene family analysis across 27 representative bird species spanning 11 avian orders. We hypothesized that Dnajc15, as an important regulator of avian mitochondrial metabolism, would show evidence of strong purifying selection acting to constrain its coding sequence, with weak to moderate codon bias and only limited evidence of adaptive change at specific sites. The gene family analysis was designed to examine whether the broader pattern of Dnajc15 retention across vertebrates is consistent with long-term functional constraint. Together, these analyses provide a comparative characterization of Dnajc15 evolution in birds and identify candidate sites for future functional investigation.

## 2 Materials and methods

### 2.1 Sequence data retrieval and alignment

*Dnajc15* coding sequences from 27 representative bird species were acquired from NCBI (Supplementary Table S1). Species were selected to represent 11 avian orders, including Passeriformes (11 species), Galliformes (2 species), Anseriformes (3 species), Accipitriformes (2 species), Falconiformes (2 species), Apterygiformes (2 species), and five additional orders with one representative each, ensuring broad phylogenetic coverage of extant bird diversity including both volant and flightless lineages. All sequences were confirmed to encode complete open reading frames prior to alignment. Multiple sequence alignments were performed using the ClustalW algorithm implemented in MEGA11 (Tamura et al., 2021), followed by manual inspection and refinement in BioEdit 7.2.1 (Hall, 1999) to remove ambiguously aligned positions. The resulting alignment contained no internal gap characters, confirming that reading frame integrity was maintained throughout.

### 2.2 Base composition and amino acid usage

Nucleotide composition was calculated using DAMBE v7.0 (Xia, 2018). This analysis also provided total AT and GC content (%), including overall base frequencies and the frequencies of each nucleotide at the first, second, and third codon positions. Overall AT and GC contents (%) were also recorded. Nucleotide compositional asymmetry was assessed using xy skew calculated as: xy skew = (x − y)/(x + y) (Chakraborty et al., 2019), where positive values indicate higher abundance of x, negative values indicate higher abundance of y and zero indicates equal usage (Perna and Kocher, 1995). CDS sequences were translated into peptide sequences using TBtools v2.0 (Chen et al., 2020) and amino acid composition was then determined from the translated sequences.

### 2.3 Codon usage indices

Codon usage indices were calculated using CodonW v1.4.4 (http://codonw.sourceforge.net), including the effective number of codons (ENC), relative synonymous codon usage (RSCU), GC content at synonymous third codon positions (GC3s), and the grand average of hydropathicity (GRAVY) and aromaticity (AROMO) indices. ENC ranges from 20 (extreme bias) to 61 (no bias); an ENC ≤35 indicates significant codon usage bias (Comeron and Aguadé, 1998). RSCU = 1 indicates no bias; RSCU >1.6 indicates overrepresented codons; RSCU <0.6 indicates underrepresented codons (Sharp and Li, 1987; Wilson, 2009; Yang et al., 2021).

### 2.4 Correspondence analysis

Correspondence analysis (COA) was employed to examine variation in codon usage patterns among species. Each species was represented by its relative synonymous codon usage (RSCU) values in 59-dimensional space defined by all synonymous sense codons, excluding AUG (Met), UGG (Trp), and stop codons. Principal axes were extracted to identify the major sources of variation in codon usage among species (Suzuki et al., 2008).

### 2.5 Graphical analysis

PR2-bias analysis was used to assess departures from parity between complementary nucleotides at fourfold degenerate sites and thereby to infer the relative influence of mutational and selective processes on codon usage (Yang et al., 2021). AT-bias [A3/(A3+T3)|4] and GC-bias [G3/(G3+C3)|4] were plotted on the vertical and horizontal axes respectively, where “|4” denotes fourfold degenerate codon families. The central point (0.5, 0.5) represents equal usage of A versus T and G versus C at these sites, whereas deviations from this point indicate compositional bias (Sueoka, 1999). The ENC plot uses ENC as the vertical coordinate and GC3s as the horizontal coordinate. An expected curve was calculated (Wright, 1990) as:

$$ENC=2+GC3s+\frac{29}{\left[GC3s^{2}+{\left(1-GC3s\right)}^{2}\right]}$$

Genes near the expected curve are generally interpreted as showing codon usage patterns largely consistent with compositional constraints; genes below the curve suggest that additional factors may contribute to codon bias, such as would arise from natural selection.

Neutrality plot analysis used GC3s as the horizontal coordinate and GC12 (defined as the mean of GC1 and GC2) as the vertical coordinate (Sueoka, 1988). The regression coefficient (slope) of GC12 on GC3s was used as an indicator of the relative extent to which codon usage variation may reflect mutational pressure versus non-neutral constraints: a regression coefficient near one is generally interpreted as consistent with a stronger contribution of mutation, while a coefficient near 0 suggests a greater role for selective or other constraining forces (Jia et al., 2015).

### 2.6 Statistical analysis

Pairwise relationships among codon usage and compositional parameters were evaluated using Pearson’s or Spearman’s correlation tests, as appropriate. Pearson’s correlation was used for approximately normally distributed variables with linear relationships, whereas Spearman’s rank correlation was used when these assumptions were not met. All tests were two-tailed. A significance threshold of *p* < 0.05 was applied throughout.

### 2.7 Selection pressure analysis

The ratio of non-synonymous to synonymous substitution rates (ω = dN/dS) was used to infer the mode of selection: ω = 1 indicates neutral evolution, ω < 1 indicates purifying selection, and ω > 1 indicates positive selection. The phylogenetic topology used in all selection pressure analyses was derived from Prum et al. (2015), a comprehensive avian phylogeny based on targeted next-generation DNA sequencing of 198 bird species. This topology was adopted directly to avoid introducing phylogenetic uncertainty from *de novo* reconstruction based on a short single-gene alignment.

PAML v4 (Yang, 2007) was used to implement three classes of codon-based maximum likelihood models—branch models, site models, and branch-site models—using the CodeML procedure with the standard genetic code. The following nested model comparisons were performed: (i) one-ratio model (M0) vs. free-ratio model (FR) to test for among-branch rate variation; (ii) M0 vs. M3 (discrete) to test for among-site rate variation; (iii) M1a (nearly neutral) vs. M2a (positive selection) and M7 (beta) vs. M8 (beta + ω) to test for positive selection at the site level; (iv) branch-site model A vs. its null model (branch-site model A with ω2 = 1) for each foreground branch to test for episodic positive selection. Statistical significance was assessed using the likelihood ratio test (LRT), with 2ΔlnL compared against a chi-square distribution using degrees of freedom equal to the difference in the number of free parameters between nested models. Candidate positively selected sites were identified using the Bayes Empirical Bayes (BEB) approach with a posterior probability threshold of >0.80 (Yang et al., 2005). Because BEB support at this threshold is suggestive rather than definitive, sites identified in this way were treated as candidate sites for further investigation. To assess robustness to phylogenetic uncertainty, all CodeML analyses were repeated using the Stiller et al. (2024) family-level genome phylogeny (218 avian families), pruned to the 27 study species using R ape v5.8 (Paradis and Schliep, 2019). The primary topological difference concerns the placement of Falconiformes: sister to Passeriformes (Prum et al., 2015) versus branching independently within Australaves closer to Piciformes (Stiller et al., 2024; McTavish et al., 2025). Multiple testing correction for the five branch-site LRT p-values was performed using the Benjamini and Hochberg (1995) false discovery rate method; BH-adjusted q-values are reported in Supplementary Tables S2–S4. Full site model parameter estimates are provided in Supplementary Table S2; likelihood ratio test results are provided in Supplementary Table S3.

### 2.8 Gene family analysis

Genome assembly and gene annotation files for the great tit (*Parus major*) were obtained from NCBI. CDS sequences were extracted and translated into protein sequences. Potential *Dnajc* genes were identified using an HMM profile for the DnaJ domain from the Pfam database (http://pfam.xfam.org/), followed by structural domain analysis using SMART (http://smart.embl-heidelberg.de/) and CDD (https://www.ncbi.nlm.nih.gov/Structure/cdd/cdd.shtml). Exon-intron boundaries were determined by comparing transcriptome data with the great tit genome. Gene and protein structures were visualized using TBtools (Chen et al., 2020; Chen et al., 2022). Multiple sequence alignments were performed using ClustalX, and phylogenetic trees were constructed using MEGA11. Gene family expansion and contraction across representative vertebrate species—including the 27 bird species used in codon usage analysis plus selected non-avian vertebrate outgroups—were analyzed using the Gene Lost/Gain Analysis plugin in TBtools (Chen et al., 2020; Song et al., 2024).

## 3 Results

### 3.1 Nucleotide composition and amino acid usage

The *Dnajc15* coding sequence in most bird species spans 444 bp, encoding 148 amino acids. The average AT content (51.54% ± 0.87%) was slightly higher than the average GC content (48.46% ± 0.87%) and both GC-skew (0.10 ± 0.02) and AT-skew (0.14 ± 0.02) were positive, indicating asymmetric usage of complementary nucleotides across species.

The overall GC content exhibited a strong positive correlation with GC3s (39.57% ± 1.22%; r = 0.722, *p* < 0.01), indicating that synonymous site composition closely tracks overall sequence GC content across bird species. Consistent with this, significant positive correlations were observed between each overall nucleotide frequency and its corresponding value at the third codon position (A vs. A3: r = 0.786; T vs. T3: r = 0.816; C vs. C3: r = 0.877; G vs. G3: r = 0.735; all *p* < 0.01), suggesting that compositional variation among bird species is predominantly driven by synonymous substitutions at the third codon position or, more cautiously, is strongly reflected at the third codon position.

**FIGURE 1:**
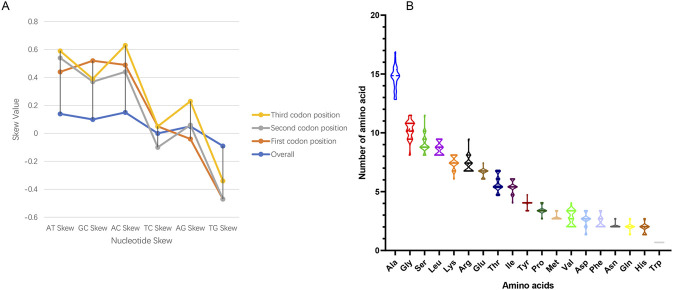
Nucleotide composition and amino acid usage patterns of avian *Dnajc15*. **(A)** Comparison of nucleotide skew values calculated from the overall coding sequence and separately from the first, second, and third codon positions; **(B)** Distribution of amino acid frequencies in the deduced *Dnajc15* protein across the sampled bird species.

Nucleotide skewness analysis revealed positive values for AT-skew, GC-skew, AG-skew, and AC-skew, while TG-skew was negative (Figure 1A), resulting in an overall nucleotide abundance order of A > G > T > C. Skewness values at the first codon position (AT1-skew, GC1-skew, AC1-skew) were consistently higher than those at the second and third positions, suggesting that nucleotide compositional asymmetry is not uniformly distributed across codon positions in *Dnajc15*. Furthermore, total GC content showed strong negative correlations with AC-skew (r = −0.931, *p* < 0.01), and strong correlations with AG-skew and TG-skew (|r| > 0.8, *p* < 0.01), indicating close associations between overall GC content and nucleotide compositional asymmetry.

Analysis of amino acid composition revealed that Ala was the most abundant residue (14.68% ± 1.04%), followed by Gly, Ser, Leu, Lys, Arg, Glu, and Thr, while His and Trp were least represented (Figure 1B). The usage frequencies of functionally similar pairs—Gly/Ser, Leu/Lys, and Asn/Gln—were not significantly different from each other, although the functional significance of these similarities remains unclear. Notably, a strong negative correlation was observed between the usage frequencies of hydrophobic Ala and hydrophilic Thr (r = −0.802, *p* < 0.01) suggesting a compositional trade-off between these two residues across bird lineages, although the basis of this pattern cannot be determined from these data alone.

Overall, *Dnajc15* shows a mildly AT-rich composition, correlated variation between overall nucleotide content and third-position composition, and non-uniform skewness across codon positions. These patterns provide descriptive context for the codon-usage analyses presented below.

### 3.2 Codon usage preferences and factors influencing *Dnajc15* in birds

*Dnajc15* exhibited weak to moderate codon usage bias across bird species, as indicated by ENC values ranging from 51.72 to 61.00 (mean: 55.97 ± 2.82). The White-headed Sea Eagle, Okarito Brown Kiwi, and Brown Kiwi showed the highest ENC values (61.00), indicating near-equal usage of all synonymous codons with minimal bias. The Brown-backed Snowfinch had the lowest ENC value (51.72), reflecting the strongest codon preference among the species examined. A significant positive correlation was observed between ENC values and AT-skew at the first codon position (*p* < 0.01), suggesting that species with greater AT compositional asymmetry at the first codon position tend to exhibit weaker overall codon bias.

Analysis of RSCU values across 59 sense codons revealed a range from 0.048 (AGC) to 2.455 (GUU), with a pronounced prevalence of A/U-ending codons among the preferred synonymous codons (Table 1). Seven codons showed strong overrepresentation (RSCU >1.6), including GUU (Val, 2.455), CCU (Pro, 2.268), CUU (Leu, 2.058), AGU (Ser, 1.952), CUG (Leu, 1.803), ACA (Thr, 1.695), and GCA (Ala, 1.607). Conversely, codons ending in G or C were consistently underrepresented (RSCU <0.6), including AGC (Ser, 0.048), CCC (Pro, 0.094), and CUA (Leu, 0.000). Overall, preferred codons were more often A/U-ending than G/C-ending, consistent with the mildly AT-rich composition described earlier.

**TABLE 1:** Codon usage bias of Dnajc15 across bird species reveals a preference for A/U-ending codons.

Codon	AA	Numbers	RSCU
GCU†	Ala	73	0.536
GCG†	Ala	36	0.264
GCC	Ala	217	1.593
GCA**	Ala	219	1.607
UGU†	Cys	0	0
UGC†	Cys	0	0
GAU†	Asp	25	0.526
GAC	Asp	70	1.474
GAG	Glu	132	1.082
GAA	Glu	112	0.918
UUU	Phe	63	1.432
UUC†	Phe	25	0.568
GGU	Gly	96	1.005
GGG†	Gly	46	0.482
GGC	Gly	129	1.351
GGA	Gly	111	1.162
CAC†	His	19	0.543
CAU	His	51	1.457
AUU	Ile	98	1.47
AUA	Ile	50	0.75
AUC	Ile	52	0.78
AAA	Lys	206	1.487
AAG†	Lys	71	0.513
CUA†	Leu	0	0
CUC†	Leu	6	0.139
CUG**	Leu	78	1.803
CUU**	Leu	89	2.058
UUA	Leu	52	0.707
UUG	Leu	95	1.293
AUG	Met	105	1
AAC†	Asn	20	0.513
AAU	Asn	58	1.487
CCA	Pro	44	1.386
CCC†	Pro	3	0.094
CCU**	Pro	72	2.268
CCG†	Pro	8	0.252
CAA	Gln	33	0.868
CAG	Gln	43	1.132
AGA	Arg	73	0.954
AGG	Arg	80	1.046
CGA	Arg	33	1.109
CGC	Arg	33	1.109
CGG	Arg	26	0.874
CGU	Arg	27	0.908
AGC†	Ser	3	0.048
AGU**	Ser	122	1.952
UCA	Ser	76	1.427
UCC	Ser	57	1.07
UCG†	Ser	17	0.319
UCU	Ser	63	1.183
ACC†	Thr	27	0.514
ACA**	Thr	89	1.695
ACG†	Thr	25	0.476
ACU	Thr	69	1.314
GUU**	Val	62	2.455
GUG†	Val	6	0.238
GUC†	Val	7	0.277
GUA	Val	26	1.03
UGG	Trp	25	1
UAC	Tyr	93	1.265
UAU	Tyr	54	0.735

Relative synonymous codon usage (RSCU) values are shown along with amino acid identity (AA) and codon counts. Codons with RSCU >1.6 are considered overrepresented, whereas those with RSCU <0.6 are underrepresented. Codons with zero counts were not observed in the analysed Dnajc15 coding sequences.

**FIGURE 2:**
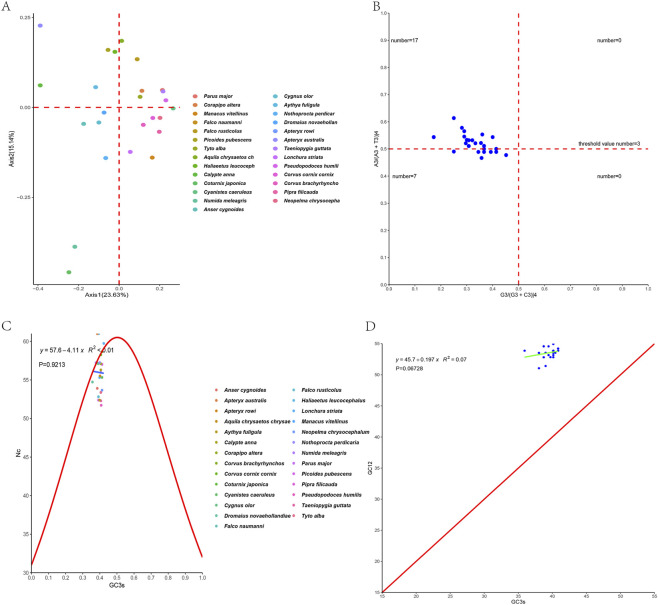
Codon-usage analyses of *Dnajc15* in birds. **(A)** Correspondence analysis (COA) based on RSCU values of 59 synonymous sense codons. The percentages on the axes indicate the proportion of total variation explained by each axis. **(B)** PR2-bias plot showing GC-bias [G3/(G3 + C3)|4] *versus* AT-bias [A3/(A3 + T3)|4], where “|4” denotes fourfold degenerate codon families and A3, T3, G3 and C3 indicate nucleotide content frequencies at the third codon position. Dashed lines at 0.5 indicate equal usage of complementary nucleotides. **(C)** ENC-GC3s plot showing the relationship between the effective number of codons (ENC) and GC content at synonymous third codon positions (GC3s). The red curve indicates the expected relationship under codon usage determined primarily by nucleotide composition: ENC = 2 + GC3s + 29/[GC3s^2^ + (1 - GC3s)^2^]. **(D)** Neutrality plot of GC3s (GC content at the third codon position) versus GC12 (average of GC1 and GC2). The fitted regression line is shown in green, and the red diagonal indicates the line of equality. The reported p-value indicates the significance of the linear relationship between GC3s and GC12.

Correspondence analysis based on RSCU values revealed that the first two principal axes explained 23.63% and 15.14% of the total variation, respectively (Figure 2A), together accounting for 38.77% of the total variation. Sample points showed lineage-associated clustering tendencies, with Passeriformes species predominantly distributed in quadrants I and IV and Galliformes concentrated in quadrant I, while flightless birds showed more dispersed distributions, although these patterns were descriptive rather than formally tested. To formally assess whether flight capability influences selective pressure on *Dnajc15*, we compared per-branch ω values from the free-ratio model between flightless (*Apteryx rowi*, *Apteryx australis*, *Dromaius novaehollandiae*; n = 3) and volant (n = 22) species using a two-sided Mann-Whitney U test. Flightless taxa showed numerically lower median ω (0.0001 vs. 0.281; U = 10, p = 0.057), a borderline non-significant result that should be interpreted with caution given the very small flightless sample (n = 3).

PR2 bias analysis, conducted using only fourfold degenerate codons (|4), revealed a mean AT-bias of 0.52 ± 0.03 and GC-bias of 0.33 ± 0.07 (Figure 2B). The majority of data points (17 of 27) fell in the upper-left quadrant (AT-bias >0.5, GC-bias <0.5), indicating preferential use of A over T and C over G at fourfold degenerate sites, collectively reflecting a dominant AC compositional preference. No species fell in the right half of the plot (GC-bias >0.5), further confirming the consistent C-over-G bias across all examined bird lineages. The deviation of both values from the neutral expectation of 0.5 suggests that codon usage in *Dnajc15* is not fully explained by parity between complementary bases at fourfold degenerate sites.

ENC-GC3s plot analysis demonstrated that GC3s values ranged from 0.357 to 0.423 and showed no significant correlation with ENC values (*p* = 0.921, Figure 2C). Critically, most data points fell below the expected curve, indicating that the observed codon bias is stronger than would be predicted by mutational pressure alone, suggesting that factors in addition to nucleotide composition, such as natural selection, may contribute to codon bias.

Neutrality plot analysis of GC3s versus GC12 revealed mean values of 39.57% ± 1.22% and 53.60% ± 0.94%, respectively, with no significant correlation between the two (*p* = 0.067, Figure 2D). Data points were narrowly distributed along both axes, reflecting limited compositional variation across species. The regression coefficient of 0.197 is consistent with a relatively limited association between GC3s and GC12 and suggests that mutation alone is unlikely to explain the observed codon-usage pattern (Jia et al., 2015).

Taken together, these analyses indicate that avian *Dnajc15* has weak overall codon bias, a preference for A/U-ending codons, and codon-usage patterns that are not fully explained by mutation alone. These results are consistent with a contribution of non-neutral constraints, which are examined further in the selection analyses below.

### 3.3 Adaptive evolutionary analysis of *Dnajc15* in birds

To investigate the selective pressures acting on *Dnajc15* across bird lineages, we applied branch, branch-site, and site models implemented in PAML v4.

**FIGURE 3:**
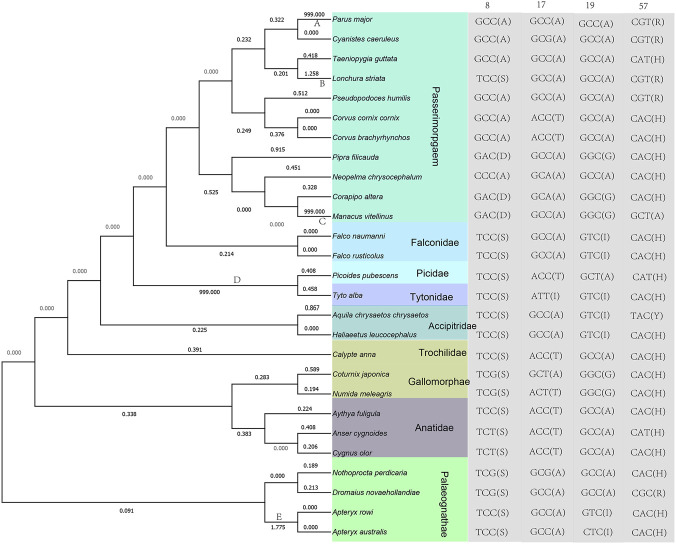
Selection analyses of *Dnajc15* across birds. Branch A phylogenetic tree showing the species included in the selection analyses and ω values for *Dnajc15* across avian lineages. The topology of the phylogenetic tree follows Prum et al. (2015). Numbers on branches are the ω values under the free-ratio model, and branches A–E indicate the foreground branches used in the two-ratio and branch-site model tests (see Table 2 for detailed results). Branch B codon and amino acid states at the four candidate sites inferred under the M8 site model (positions 8, 17, 19, and 57).

Under the branch model, the free-ratio model did not significantly differ from the one-ratio model (2ΔlnL = 35.1, df = 42, *p* = 0.77, Table 2), suggesting that *Dnajc15* has experienced broadly similar selective pressures across most bird lineages. The overall ω estimate of 0.33 under the one-ratio model indicates that the gene is predominantly subject to strong purifying selection, consistent with substantial functional constraint. Nevertheless, five specific branches (A, B, C, D and E in Figure 3) exhibited ω values exceeding one under the free-ratio model, suggesting either relaxed constraint or possible lineage-specific positive selection in these lineages. Two-ratio model comparisons (MA–ME) did not yield significant LRT values for any of the tested foreground branches (*p* = 0.06–0.47, Table 2), indicating that elevated ω values in these branches do not reach statistical significance when tested individually. Branch-site model analyses similarly failed to identify significant positive selection on any specific foreground branch (branches A, B, C, D and E, Table 2). Accordingly, branch-site results do not provide robust support for lineage-specific positive selection in the sampled bird lineages. After Benjamini–Hochberg false discovery rate correction of the five branch-site LRT p-values, all branches returned adjusted q-values of 1.00 under both the Prum 2015 and Stiller 2024 topologies (Supplementary Table S4), further confirming the absence of lineage-specific positive selection (Supplementary Tables S2 and S3).

**TABLE 2:** Selection analyses of *Dnajc15* across the bird species analysed in this study.

Model category	Model	-InL	Parameters	Comparison	d.f	2ΔInL	*p* value	Positive site (BEB)
Branch model	One ratio (M0)	2931.43	ω = 0.32787	M0&FR	42	35.1	0.77	none
​	Free ratio (FR)	2913.89	ω variation for each branch	​	​	​	​	​
​	Two ratios	​	​	​	​	​	​	​
​	MA	2930.65	ω0 = 0.33, ω1 = 999.00	M0&MA	1	1.55	0.21	none
​	MB	2930.69	ω0 = 0.32, ω1 = 1.29	M0&MB	1	1.47	0.23	none
​	MC	2927.97	ω0 = 0.33, ω1 = 999.00	M0&MC	1	3.46	0.06	none
​	MD	2931.16	ω0 = 0.33, ω1 = 999.00	M0&MD	1	0.53	0.47	none
​	ME	2930.25	ω0 = 0.32, ω1 = 1.46	M0&ME	1	2.36	0.12	none
Branch-site model	A (Ma)	2891.84	ω0 = 0.09, ω1 = 1.00, ω2 = 999.00	A (Ma0&Ma)	1	0.21	0.65	none
​	A (Ma0)	2891.74	ω0 = 0.09, ω1 = 1.00, ω2 = 1.00	​	​	​	​	​
​	B (Ma)	2891.12	ω0 = 0.09, ω1 = 1.00, ω2 = 3.91	B (Ma0&Ma)	1	0.19	0.66	none
​	B (Ma0)	2891.21	ω0 = 0.09, ω1 = 1.00, ω2 = 1.00	​	​	​	​	​
​	C (Ma)	2885.66	ω0 = 0.08, ω1 = 1.00, ω2 = 999.00	C (Ma0&Ma)	1	1.18	0.28	none
​	C (Ma0)	2886.25	ω0 = 0.08, ω1 = 1.00, ω2 = 1.00	​	​	​	​	​
​	D (Ma)	2891.74	ω0 = 0.33, ω1 = 1.00, ω2 = 1.00	D (Ma0&Ma)	1	79.37	5.14E-19	none
​	D (Ma0)	2931.43	ω0 = 0.09, ω1 = 1.00, ω2 = 1.00	​	​	​	​	​
​	E (Ma)	2891.71	ω0 = 0.09, ω1 = 1.00, ω2 = 4.59	E (Ma0&Ma)	1	0.07	0.79	none
​	E (Ma0)	2891.74	ω0 = 0.09, ω1 = 1.00, ω2 = 1.00	​	​	​	​	​
Sites model	M3 (discrete)	2889.14	p0 = 0.08, ω0 = 0.01 p1 = 0.67, ω1 = 0.13 p2 = 0.25, ω2 = 1.13	M0&M3	4	84.58	1.86E-17	5 A (0.947); 6 A (0.942); 8 A (1.000**); 10 S (0.952*); 13 Y (0.817); 17 A (1.000**); 19 A (1.000**); 22 G (0.994**); 25 K (0.999**); 27 Q (0.839); 28 A (0.999**); 30 L (0.921); 41 L (0.906); 48 F (0.884); 57 R (1.000**); 58 V (0.960*); 65 T (0.980*); 66 I (0.897); 75 T (0.873); 78 F (0.955*); 80 S (0.961*); 106 G (0.995**); 108 D (0.993**); 112 T (0.977*); 116 K (0.980*); 141 L (0.906)
​	M1a (nearly neutral)	2891.74	P0 = 0.71117, ω0 = 0.09149, P1 = 0.28883, ω1 = 1.00000	M1a&M2a	1	0	1	​
​	M2a (positive selection)	2891.74	P0 = 0.96, ω0 = 0.01, p1 = 0.04, ω1 = 1.00, p2 = 0.00, ω2 = 22.19	​	​	​	​	8 A (0.708); 17 A (0.689); 19 A (0.721); 57 R (0.687)
​	M7 (beta)	2898.46	p = 0.64606, q = 1.28149	M7&M8	2	16.4	0.000274	​
​	M8 (beta&ω)	2890.26	p0 = 0.78876, p = 1.35829, q = 7.96415, p1 = 0.21124, ω = 1.21867	​	​	​	​	8 A (0.907); 17 A (0.911); 19 A (0.921); 25 K (0.539); 28 A (0.635); 57 R (0.898)
​	M8a (beta&ω = 1)	2891.05	p0 = 0.73535, p = 1.53204, q = 11.82575, p1 = 0.26465, ω = 1.00000	​	​	​	​	​

For each model, the negative log-likelihood (−lnL), estimated parameters, model comparison, degrees of freedom (d.f.), likelihood-ratio statistic (2ΔlnL), P-value, and candidate sites identified by Bayes Empirical Bayes (BEB) are shown where applicable. ω denotes dN/dS. Sites with BEB posterior probabilities >0.80 are reported as candidate sites for further investigation. Asterisks in the site-model rows denote BEB support levels from the original output rather than independent significance tests. For Branch D, D (Ma0) converged to the same likelihood as M0 (−lnL = 2931.43), suggesting optimization failure due to the extremely short foreground branch. The LRT statistic is therefore unreliable and not interpreted.

**FIGURE 4:**
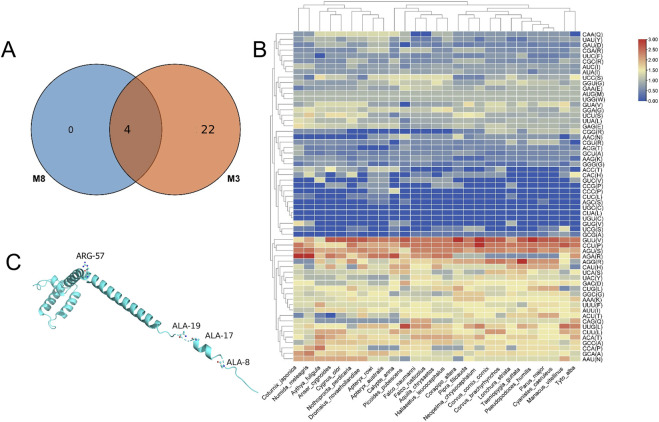
Candidate sites and codon-usage patterns of *Dnajc15* across birds. **(A)** Venn diagram showing the overlap between candidate sites identified by the M3 and M8 site models. **(B)** Heat map of the RSCU values for *Dnajc15* across the sampled bird species. The horizontal axis represents hierarchical clustering of bird species and the vertical axis represents hierarchical clustering of codons based on RSCU values. RSCU values less than 0.6 indicate underrepresentation, whereas values greater than 1.6 indicate overrepresentation. **(C)** Three-dimensional structural model of the *Dnajc15* monomer showing the positions of the four candidate sites inferred under the M8 site model.

At the gene-wide level, site model analyses provided evidence of among-site heterogeneity and limited support for positive selection at specific codon positions. The M0 vs. M3 comparison confirmed significant heterogeneity in ω values across sites (2ΔlnL = 84.58, df = 4, *p* = 1.86 × 10^−17^, Table 2), indicating that different regions of the gene evolve at markedly different rates. The M7 vs. M8 comparison yielded a significant LRT (2ΔlnL = 16.4, df = 2, *p* = 0.00027), with M8 identifying six candidate sites with ω > 1, four of which had posterior probabilities exceeding 0.80 by the Bayes Empirical Bayes (BEB) method: positions 8 (Ala, BEB = 0.907), 17 (Ala, BEB = 0.911), 19 (Ala, BEB = 0.921), and 57 (Arg, BEB = 0.898). However, the M1a vs. M2a comparison was not significant (2ΔlnL = 0, df = 1, *p* = 1.00), indicating that the positive selection model did not provide a better fit than the nearly neutral model under this test. The discordance between M7 vs. M8 and M1a vs. M2a results is consistent with weak or episodic positive selection at a small number of sites, a pattern that is known to be more readily detected by the beta-distribution-based M7/M8 framework than by M1a/M2a in datasets with limited taxon sampling (Anisimova et al., 2002). The Venn diagram (Figure 4A) illustrates the overlap between candidate sites identified by M3 and M8.

The four candidate positive selection sites identified by M8 are of particular biological interest (Figures 3B, 4C). Positions 8, 17, and 19 are located in the N-terminal region of the MCJ protein, in close proximity to the highly conserved “LRYAEY” peptide motif present across birds, reptiles, mammals, and fish. Notably, Passeriformes species consistently carry alanine at position 8, whereas non-Passeriformes birds predominantly display serine at this position, indicating lineage-associated amino acid differentiation. Position 57 falls within the J-domain and may influence the chaperone function of MCJ. These sites should be regarded as candidates for future functional investigation to determine their significance for mitochondrial energy regulation.

Taken together, the selection pressure analysis indicates that *Dnajc15* is predominantly conserved under purifying selection across bird lineages, with preliminary evidence for positive selection at four specific sites. These findings are consistent with the functional importance of the MCJ protein and suggest that adaptive evolution, where present, is restricted to functionally important regions of the protein.

### 3.4 Evolutionary trends and variation in the *dnajc* gene family across species

Structural domain analysis of the great tit (*P. major*) genome identified *Dnajc15* as a vertebrate duplication of *Dnajc19*. Compared to *Dnajc19*, *Dnajc15* lacks the N-terminal region present in the ancestral gene, indicating structural divergence and possible functional divergence following gene duplication.

**FIGURE 5:**
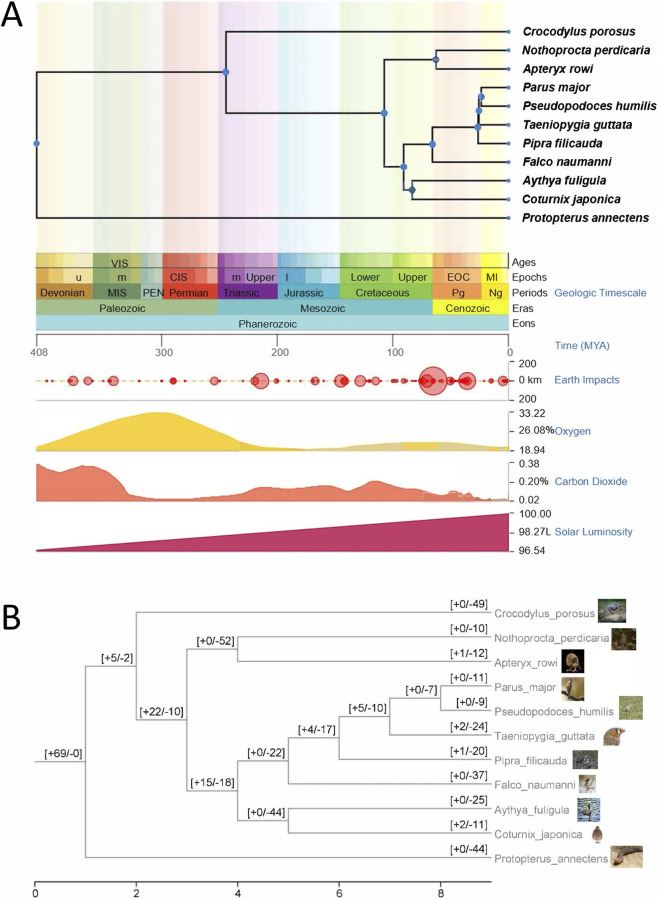
Evolutionary context and gene-family dynamics of *Dnajc* across representative vertebrates. **(A)** Species divergence timeline shown alongside the geological timescale and selected environmental variables, including atmospheric oxygen and CO_2_ concentrations and solar luminosity, to provide temporal context for the observed phylogenetic relationships. These variables are included for contextual reference only; no formal analysis of their relationship with *Dnajc* gene family evolution was conducted in the present study. **(B)** Gene family expansion and contraction inferred for the *Dnajc* gene family across representative vertebrate species. Values in brackets indicate inferred gene gains (+) and losses (−) along each branch.

Gene family expansion and contraction analysis revealed broad patterns of gain and loss in the *Dnajc* gene family across representative vertebrate species (Figure 5B). A large expansion ([+69/-0]) was inferred at the root node, suggesting that the *Dnajc* gene family underwent substantial diversification early in vertebrate evolution. Across all examined terminal lineages, contraction was the predominant pattern. Among non-avian outgroups, the Australian saltwater crocodile (*Crocodylus porosus*, [+0/-49]) and the West African lungfish (*Protopterus annectens*, [+0/-44]) showed the most extensive gene loss. Among birds, all examined species exhibited net contraction at their terminal branches regardless of taxonomic order, including Passeriformes species such as *Taeniopygia guttata* ([+2/-24]) and *Pipra filicauda* ([+1/-20]). These expansion and contraction values are based on the Gene Loss/Gain analysis implemented in TBtools and represent descriptive patterns; formal statistical significance testing of individual events was not conducted in the present study.

Across this background of pervasive gene family contraction, *Dnajc15* was retained in all examined species. This pattern of universal retention is consistent with the strong purifying selection identified in our selection pressure analysis (ω = 0.33), and suggests that *Dnajc15* is under stronger long-term evolutionary constraint than many other members of the gene family. The co-occurrence of broad *Dnajc* family contraction and strict *Dnajc15* retention across divergent vertebrate lineages—from lungfish to passerine birds—is consistent with long-term conservation of this gene and may reflect functional importance in vertebrate mitochondrial biology. Figure 5A presents the species divergence timeline alongside the geological timescale and selected environmental parameters including atmospheric oxygen and CO_2_ concentrations and solar luminosity, providing temporal context for the observed phylogenetic relationships. These variables are shown for contextual purposes only; no formal analysis of their relationship with *Dnajc* gene family evolution was conducted in the present study.

## 4 Discussion

### 4.1 Codon usage bias and its determinants in avian *Dnajc15*

The MCJ protein encoded by *Dnajc15* acts as an endogenous repressor of the mitochondrial respiratory chain and plays roles in mitochondrial biogenesis and fertilization (Hatle et al., 2013). As a conserved member of the HSP40 family, *Dnajc15* provides a tractable model for examining the interplay between functional constraint and sequence evolution in birds.

The weak codon usage bias observed in avian *Dnajc15* (ENC: 51.72–61.00) is consistent with relatively limited overall codon bias in this gene across the sampled bird species. In general, genes subject to strong translational selection—typically those that are highly expressed—tend to display lower ENC values and stronger codon bias (Chakraborty et al., 2019; Uddin and Chakraborty, 2016). The high ENC values observed here suggest that *Dnajc15* is not under strong translational selection for expression speed or accuracy, which may reflect its function as a regulatory rather than a constitutively high-output protein. The weak but consistent preference for A/U-ending codons across all 27 species further indicates a shared compositional tendency that is consistent with the AT-rich background of avian *Dnajc15* sequences.

Our neutrality plot and ENC analyses are consistent with the mutation-selection-drift model of codon usage evolution, and suggest that mutational pressure alone is unlikely to explain the observed codon-usage patterns. These results are therefore consistent with a contribution of non-neutral constraints to codon usage in this gene. The lineage-associated clustering observed in COA analysis—with Passeriformes and Galliformes showing distinct distributions—suggests that codon-usage patterns can vary among lineages even in the absence of changes in encoded amino acid sequences. Notably, flightless birds showed more dispersed COA distributions compared to volant species, potentially reflecting relaxed or altered selective pressures associated with the loss of the metabolically demanding flight function. Per-branch ω values from the free-ratio model provide a complementary perspective: *Calypte anna* (hummingbird) exhibited ω = 0.429, while *A*. *rowi* and *A. australis* showed ω = 0.0001 and *D*. *novaehollandiae* showed ω = 0.091. A formal Mann-Whitney U test comparing flightless (n = 3) and volant (n = 22) species yielded a borderline non-significant result (U = 10, p = 0.057), which should be interpreted with caution given the limited flightless sample. The relationship between codon usage bias and natural selection is further complicated by potential non-adaptive nucleotide composition biases that can confound estimates of selection on synonymous codon usage (Cope and Shah, 2022; LaBella et al., 2019).

Compared to the codon usage patterns reported for *HSP60* in birds (Yang et al., 2021), *Dnajc15* shows broadly similar AT-ending codon preferences and a comparable signal that codon usage is not fully explained by mutation alone; selection is also likely involved. However, *Dnajc15* displays higher ENC values overall, indicating weaker overall codon bias than reported for HSP60. This comparison suggests that while the general framework of codon usage evolution is conserved across HSP family members in birds, gene-specific functional and structural features modulate the strength and pattern of codon bias.

### 4.2 Selective pressures and candidate adaptive sites

The strong purifying selection acting on *Dnajc15* (overall ω = 0.33, with 68% of sites showing ω < 1) is consistent with the functionally important and strongly constrained role of the MCJ protein in mitochondrial respiratory chain regulation. This pattern indicates that amino acid-changing substitutions are generally disfavoured across much of the coding sequence. The overall ω estimate of 0.33 falls within the typical range for vertebrate protein-coding genes (Wolf et al., 2009); the inference of functional constraint rests on the convergence of multiple lines of evidence—universal single-copy retention across all 27 species, absence of robust positive selection across site and branch-site models under both tree topologies, and deep conservation of Dnajc15 orthologues across vertebrates.

The four candidate positively selected sites identified by the M7 vs. M8 comparison (positions 8, 17, 19, and 57) are of particular interest given their locations in functionally important protein regions. Positions 8, 17, and 19 cluster in the N-terminal region of MCJ, adjacent to the highly conserved “LRYAEY” motif present across vertebrate lineages. The lineage-specific substitution at position 8 — where Passeriformes consistently carry alanine while non-Passeriformes display serine—is notable given the physicochemical difference between these residues and the proximity of this site to the conserved peptide motif. Position 57, located within the J-domain, may influence the chaperone function of MCJ through its interaction with HSP70 (Jiang et al., 2019). However, it should be emphasized that the positive selection signal at these sites was supported by M7 vs. M8 but not corroborated by M1a vs. M2a (The Ala→Ser substitution at site 8 carries a Grantham (1974) distance score of 99, reflecting a moderate-to-radical physicochemical change from non-polar to polar residue. At site 19, the Gly→Ile substitution (score = 135) involves a substantial increase in side-chain volume and hydrophobicity. The Arg→His change at site 57 (score = 29) is conservative (both basic); the Arg→Ala change in Manacus vitellinus (score = 112) involves loss of positive charge. *P* = 1.00), indicating weak or episodic rather than pervasive positive selection. These sites therefore represent candidates for future functional investigation rather than confirmed adaptive loci.

The branch-specific analyses did not identify significant positive selection on any foreground branch, suggesting that adaptive evolution in *Dnajc15* is restricted to a small number of specific sites rather than being lineage-driven. This pattern is consistent with a model in which the gene is globally constrained by purifying selection, with only limited sites retaining the capacity for adaptive change—likely reflecting the structural and functional sensitivity of the MCJ protein to amino acid substitution.

### 4.3 Gene family dynamics and the importance of *Dnajc15*

The gene family analysis provides a macroevolutionary perspective that complements and reinforces the molecular-level findings. The pervasive contraction of *Dnajc* family members across all examined vertebrate lineages—including both birds and non-avian outgroups—stands in contrast to the strict retention of *Dnajc15* in every species examined. This pattern of selective retention against a background of widespread gene loss is consistent with the inference that *Dnajc15* has been subject to stronger long-term evolutionary constraint than many other members of the gene family (Xiong et al., 2023).

The origin of Dnajc15 orthologues are also conserved as single-copy genes across non-avian vertebrates—including mammals, squamate reptiles, and teleost fishes—demonstrating deep conservation consistent with maintained functional constraint since the origin of vertebrates (Li et al., 2017; Xiong et al., 2010; Dong et al., 2019). *Dnajc15* as a vertebrate duplication of *Dnajc19*, accompanied by loss of the N-terminal region, suggests that functional specialization occurred at the time of duplication. The subsequent conservation of *Dnajc15* across divergent vertebrate lineages, from lungfish to passerine birds, implies that this specialized function—regulation of Complex I activity in the mitochondrial respiratory chain—has been retained under strong purifying selection over long evolutionary timescales. Taken together, the codon usage, selection pressure, and gene family analyses converge on the same conclusion: *Dnajc15* is a functionally important component of the vertebrate mitochondrial regulatory network.

### 4.4 Comparative perspectives and future directions

While this study focuses on avian *Dnajc15*, comparative evidence suggests that the gene plays important and conserved roles in mammalian mitochondrial function, including roles in cancer suppression and metabolic regulation (Hatle et al., 2013; Cicuéndez et al., 2025). The strong purifying selection observed in birds may also characterize mammalian lineages, though differences in metabolic demands—particularly the high energy turnover associated with avian flight—may modulate the strength and pattern of selection at specific sites. Whether the candidate positive selection sites identified here in birds show parallel patterns of adaptive evolution in metabolically demanding mammalian lineages, such as bats or small rodents, represents an open and tractable question for future study. Bats (order Chiroptera), as the only mammals capable of powered flight, represent a compelling independent comparative system; examination of Dnajc15 evolution across chiropteran lineages would provide an independent test of whether flight-associated metabolic demands drive convergent patterns of selection on this gene.

### 4.5 Limitations

Several limitations of the present study should be acknowledged. First, the relatively small sample of 27 bird species limits the statistical power of both codon usage and selection pressure analyses, and may not fully capture the diversity of evolutionary patterns across the approximately 10,000 extant avian species. Second, the short coding sequence of *Dnajc15* (444 bp, 148 amino acids) imposes inherent constraints on the resolution of PAML-based analyses, and the candidate positive selection sites identified here should be interpreted with appropriate caution. Third, the absence of gene expression data prevents direct testing of the hypothesis that weak codon bias is associated with the expression profile of *Dnajc15*. Fourth, the expansion and contraction values reported in the gene family analysis are descriptive and were not subjected to formal statistical significance testing. Future studies incorporating broader taxonomic sampling, expression profiling, and functional mutagenesis of candidate sites would substantially strengthen inference regarding codon-usage evolution, selective constraint, and the functional significance of the candidate sites identified in this study. Formal statistical evaluation of gene family evolution rates using CAFE (Han et al., 2013) would require a time-calibrated ultrametric tree with branch lengths, representing a direction for future work. Fifth, the short alignment (444 bp) and limited taxon sampling (n = 27) constrain the statistical power of PAML-based tests; simulation-based power analyses would be needed to rigorously quantify detection limits for this dataset.

## Data Availability

The original contributions presented in the study are publicly available. The Dnajc15 coding sequences can be found in NCBI GenBank; accession numbers for all 27 species are listed in Supplementary Table S1.
